# Identification of Cause of Posttransplant Cachexia by PCR

**DOI:** 10.3201/eid1808.120309

**Published:** 2012-08

**Authors:** Joelle Guitard, Sophie Edouard, Hubert Lepidi, Christine Segonds, Marion Grare, Marie-Laure Ranty-Quintyn, Isabelle Rouquette, Olivier Cointault, Lionel Rostaing, Nassim Kamar, Florence Fenollar

**Affiliations:** Centre Hospitalier Universitaire Rangueil, Toulouse, France (J. Guitard, C. Segonds, M. Grare, M.-L. Ranty-Quintyn, I. Rouquette, O. Cointault, L. Rostaing, N. Kamar);; Université Aix-Marseille, Marseille, France (S. Edouard, H. Lepidi, F. Fenollar);; Pôle de Maladies Infectieuses, Marseille (S. Edouard, F. Fenollar);; and Université Paul Sabatier, Toulouse (L. Rostaing, N. Kamar)

**Keywords:** Mycobacterium genavense, Whipple’s disease, Tropheryma whipplei, nontuberculous mycobacterium, bacteria, solid organ transplantation

**To the Editor:** A man, 56 years of age, was admitted to the hospital for epigastric pain, fever, and fatigue 8 years after a cardiac transplant. His immunosuppressive regimen consisted of cyclosporine A, mycophenolate mofetil, and steroids. Clinical examination revealed a 4-kg weight loss within 3 months without peripheral lymph node enlargement.

Laboratory test results showed moderate anemia, severe lymphopenia, and moderately increased C-reactive protein. Serologic results for HIV, *Brucella* spp., *Coxiella burnetii*, and *Francisella tularensis* were negative. Whole-body computed tomography scanning showed enlarged mediastinal and abdominal lymph nodes. Bone marrow histopathologic results ruled out lymphoma or granuloma but showed a histiocytic infiltrate and intracellular acid-fast bacilli (AFB) with positive Ziehl–Neelsen staining. Sputum, urine, gastric aspirates, and bronchoalveolar lavage specimens revealed no AFB. A mediastinal lymph node biopsy showed few AFB, suggesting *M. tuberculosis* or nontuberculous mycobacteria. Isoniazid, rifampin, ethambutol, and clarithromycin were prescribed for 2 months, followed by rifampin, ethambutol, and clarithromycin. Cultures for mycobacteria remained negative.

Five months after treatment initiation, the patient experienced severe abdominal pain, diarrhea, and continued weight loss. Lymph node biopsy was repeated; results showed intramacrophagic coccobacilli tinted with Ziehl-Neelsen, Gram, and periodic acid–Schiff (PAS) stains. Two diagnoses were considered: malakoplakia and Whipple disease (WD). Screening results from quantitative real-time PCR (qPCR) for *Tropheryma whipplei* were negative for blood, saliva, stools, urine, and lymph nodes.

Although no characteristic Michaelis–Gutmann bodies were seen, the staining characteristics of the intracellular coccobacilli were compatible with *Rhodococcus equi*, a pathogen associated with malakoplakia. Combined treatment with ertapenem, teicoplanin, and amikacin was implemented but failed to induce clinical improvement. Culture of the biopsy specimen failed to grow *R. equi* or mycobacteria, and the result of 16S rRNA PCR was negative. To investigate the cause of the diarrhea, the patient underwent endoscopy, which showed a thickened duodenal wall. A duodenal biopsy specimen displayed a massive histiocytic infiltrate, with positive PAS and Gram staining but negative Ziehl-Neelsen staining. Cultures remained negative for mycobacteria.

Acting on the hypothesis of WD, we administered doxycycline and hydroxychloroquine for 4 weeks, then discontinued for ineffectiveness. Four weeks after cessation of antimicrobial drug treatment, a third lymph node biopsy was performed, in which the *T. whipplei* PCR result was positive. Antibacterial drug treatment for WD was resumed, but the patient’s condition worsened.

Simultaneously, extracted DNA and fresh tissue of all biopsy specimens were sent to the Unité de Recherche sur les Maladies Infectieuses et Tropicales Emergentes, (Marseille, France), a reference laboratory for WD. Immunohistochemical analysis, DNA extraction, and *T. whipplei* qPCR were performed as described (*1**,**2*). Biopsy specimens were subjected to a systematic molecular approach, which included 16S rRNA PCR and several specific PCRs (*3*) (Table A1).

Histopathologic results of the duodenal biopsy revealed PAS-positive and diastase-resistant macrophages (Figure) with faint immunohistochemical staining. Results of *T. whipplei* PCRs targeting 2 different sequences were negative for the duodenal and lymph node biopsy specimens. These specimens were also negative by PCR for 16S rRNA, *Bartonella* spp., and *F. tularensis*. Conversely, Ziehl–Neelsen staining showed numerous AFB. Results of PCRs were negative for *M. tuberculosis* and *M. avium* but positive for *Mycobacterium* spp.

**Figure:**
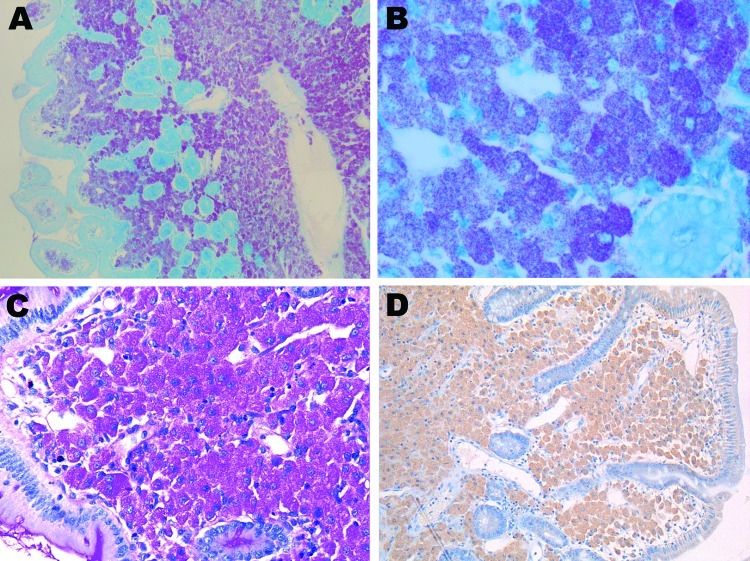
Duodenal biopsy specimen from the patient with posttransplant cachexia. Ziehl–Neelsen acid staining of a patient biopsy specimen, showing partially reduced villous architecture at low magnification, with numerous Ziehl–Neelsen-positive macrophages within the lamina propria (A, original magnification ×50). High magnification clearly demonstrates mycobacteria as bacillary particles in the macrophage cytoplasm (B, original magnification ×400). C) Macrophages within the lamina propria are periodic acid-Schiff–positive, diastase-resistant particles but do not show a morphologic granular pattern (original magnification ×200). D) Immunohistochemical staining with a polyclonal rabbit antibody against *Tropheryma whipplei* shows low immunostaining without a granular pattern (antibody used at a dilution of 1:2,000, hemalun counterstain, original magnification ×100).

Sequencing facilitated identification of *Mycobacterium genavense* (99.6% of homology with the isolate with GenBank accession no. HM022216). Combined treatment with amikacin, rifabutin, moxifloxacin, clarithromycin, and ethambutol was implemented. To enhance the chances of eradicating *M. genavense*, mycophenolate mofetil was discontinued and cyclosporine A reduced. The patient’s condition was largely unimproved; clinical improvement was observed 9 months after treatment reinitiation. Cardiac allograft function remained unaltered. Optimal duration of therapy is unknown; treatment had been ongoing for nearly 12 months at time of publication. More than the choice of antimycobacterial agents, we believe that it is the reduction in immunosuppression and the duration of therapy that eventually facilitated clinical improvement.

*M. genavense* is a slow-growing, nontuberculous mycobacterium that infects immunocompromised hosts (*4*). Only 3 cases of *M. genavense* infection in solid-organ transplant recipients have been reported (*5**–**7*). *M. genavense* has a predilection for the digestive tract, which explains the severity of the gastrointestinal symptoms (*4*). Moreover, it can mimic the endoscopic and histopathological features of WD (*8*).

In this case, the positive PAS-staining, the weak positivity of immunochemical staining for *T. whipplei*, and the false-positive results for 1 PCR temporarily delayed diagnosis. False-positive PCR results have been mainly reported when molecular diagnosis for *T. whipplei* was based on 16S rRNA PCR (*9*). Thus, positivity of a first PCR should be confirmed by using a second PCR with another target (*10*).

Bacteria responsible for lymph node enlargement are rarely isolated by culture. Molecular methods performed on lymph node biopsy specimens are useful diagnostic tools, but the common single molecular approach using 16S rRNA PCR lacks sensitivity, which delayed diagnosis for this patient (*3*). To address this issue, simultaneously to performing 16S rRNA PCR, we followed a strategy of systematic qPCR for lymph node specimens that targeted *Bartonella* spp., *F. tularensis*, *T. whipplei*, and *Mycobacterium* spp (*3*). This report confirms the power of this systematic molecular approach, which enabled us to identify a rare bacterial agent scarcely reported for transplant patients.

## Appendix

**Table A1:** Approach used to determine the cause of posttransplant cachexia in a patient*

Pathogen	Sequence target	Primers, 5′ → 3′	Probes/identification
Molecular tool to detect and identify *Tropheryma whipplei*			
Real-time PCR			
* T. whipplei*	Repeated sequence	Twhi2F: TGAGGATGTATCTGTGTATGGGACA	6-FAM-GAGAGATGGGGTGCAGGACAGGG-TAMRA
		Twhi2R: TCCTGTTACAAGCAGTACAAAACAAA	
* T. whipplei*	Repeated sequence	Twhi3F: TTGTGTATTTGGTATTAGATGAAACAG	6-FAM-GGGATAGAGCAGGAGGTGTCTGTCTGG-TAMRA
		Twhi3R: CCCTACAATATGAAACAGCCTTTG	
Molecular tools to detect and identify *Mycobacterium* spp.			
Step 1: Real-time PCR			
*Mycobacterium* spp.	ITS	ITSd: GGGTGGGGTGTGGTGTTTGA ITSr: CAAGGCATCCACCATGCGC	6-FAM-TGGATAGTGGTTGCGAGCATC-TAMRA
* M. tuberculosis*	ITS	ITSd: GGGTGGGGTGTGGTGTTTGA ITSr: CAAGGCATCCACCATGCGC	6-FAM-GCTAGCCGGCAGCGTATCCAT-TAMRA
* M. avium*	ITS	ITSd: GGGTGGGGTGTGGTGTTTGA ITSr: CAAGGCATCCACCATGCGC	6-FAM-GGCCGGCGTTCATCGAAAT-Mgb
Step 2: Classical PCR			
*Mycobacterium* spp.	*rpoB*	MycoF: GGCAAGGTCACCCCGAAGGG MycoR: AGCGGCTGCTGGGTGATCATC	Sequencing
Housekeeping gene	β-actin	ActinF: CATGCCATCCTGCATCTGGA ActinR: CCGTGGCCATCTCTTGCTCG	6-FAM-CGGGAAATCGTGCGTGACATTAAG-TAMRA

*ITS, internal transcribed spacer; *rpoB*, RNA polymerase B.
